# RGD-expressed bacterial membrane-derived nanovesicles enhance cancer therapy *via* multiple tumorous targeting

**DOI:** 10.7150/thno.51988

**Published:** 2021-01-15

**Authors:** Jin Gao, Sihan Wang, Xinyue Dong, Zhenjia Wang

**Affiliations:** Department of Pharmaceutical Sciences, College of Pharmaceutical Sciences and Pharmacy, Washington State University, Spokane, WA 99202, USA

**Keywords:** Bacterial membrane-derived nanovesicles, Doxorubicin, Remote loading, Tumor microenvironments, Inflammation response.

## Abstract

**Background:** A tumor microenvironment is a complicated multicellular system comprised of tumor cells, immune cells and blood vessels. Blood vessels are the barriers for drug tissue penetration. Effectively treating a cancer requires drug delivery systems to overcome biological barriers present in tumor microenvironments (TMEs).

**Methods:** We designed a drug delivery system made of bacterial (*Escherichia coli*) double layer membrane-derived nanovesicles (DMVs) with the expression of RGD peptides and endogenous targeting ligands of bacteria. The physical and biological characteristics of DMVs were assessed by cryogenic transmission electron microscopy, western blotting, flow cytometry and confocal microscopy. Doxorubicin (DOX) was loaded in DMVs via a pH gradient driven drug loading method. Therapeutical effects of DOX-loaded DMVs were studied in a melanoma xenograft mouse model.

**Results:** *In vitro and in vivo* experiments showed that DMVs can target neutrophils and monocytes that mediated the transport of DMVs across blood vessel barriers and they can also directly target tumor vasculature and tumor cells, resulting in enhanced delivery of therapeutics to TMEs. Furthermore, we developed a remote drug loading approach to efficiently encapsulate DOX inside DMVs, and the drug loading was 12% (w/w). In the B16-F10 melanoma mouse model, we showed that DOX-RGD-DMVs significantly inhibited the tumor growth compared to several controls.

**Conclusion:** Our studies reveal that DMVs are a powerful tool to simultaneously target multiple cells in TMEs, thus increasing drug delivery for improved cancer therapies.

## Introduction

To effectively treat cancers, drug delivery systems have been developed to target tumor cells *via* design of targeting ligands conjugated to nanoparticle surface 1-4. Arginine-glycine-aspartic acid (RGD) is a peptide that specifically binds fibronectin 5. The studies show that RGD 1, 6 is also a specific ligand to bind integrin α_v_β_3_, which plays a key role in tumor angiogenesis and metastasis 7. Integrin α_v_ is highly expressed on endothelial cells lining tumor blood vessels rather than on normal endothelial cells 8. Integrin α_v_β_3_ is highly expressed on various tumor cells to potentiate tumor metastasis 9, and also supports immune cells to migrate into tumor tissues 10. Therefore, targeting integrin α_v_β_3_ *via* RGD to deliver therapeutics may be a novel strategy for tumor interventions 8, 11, 12.

RGD-based targeting approaches have been intensively investigated 13-15. Several RGD peptides were developed, such as, cyclic RGD 16, RGD4C 17, RGD10 18 and RGD-peptidomimetic 19. Conjugating RGD peptides to nanoparticle surface is a major effort to improve tumor targeting and drug delivery 20. While the such strategy shows a promising therapeutic efficacy compared to free drugs 21, 22, biofunctionalization of synthetic nanoparticles requires the precise control of bioconjugation and does not achieve the predesigned tropism of nanoparticles 23, 24. Recent advances in cell membrane-derived nanovesicles demonstrate the potential to solve the challenges in conventional drug delivery systems because nanovesicles are made from cellular components with endogenous tissue targeting ligands 25-31. Bacterium-derived nanovesicles, so-called outer membrane vesicles (OMVs), have been explored as drug carriers in vaccine developments 32, 33 and cancer therapies 34. We recently reported an approach to generate double-layer membrane-derived nanovesicles (DMVs) from bacteria using nitrogen cavitation, and showed that DMVs can be used as a vaccine to combat bacterial infections 35. Compared to OMVs 36, DMVs constitute the whole membrane of gram-negative bacteria and contain endogenous cellular targeting ligands, and DMVs are also stable 26, 35, 37. Our nitrogen cavitation method can be used to generate DMVs from any bacteria and is ready to scale up for translation of our technology 27, 35. The challenges in cancer therapies using cell membrane-derived nanovesicles are how to increase tumor targeting and how to efficiently load drugs inside nanovesicles 37, 38. For example, OMVs derived from outer membrane of bacteria are complex, unstable and heterogenous, and the drug loading to OMVs is limited 34. Therefore, it is needed to create novel concepts and methods to resolve the limitations in OMVs.

Tumor microenvironments (TMEs) are a special organization to support tumor growth and metastasis, leading to patient death 39. TMEs are comprised of tumor cells, immune cells and blood vessels. Effectively treating tumors requires drug delivery systems to overcome biological barriers present in TMEs 40, 41, such as blood vessel barrier and tumor targeting.

**Figure 1:**
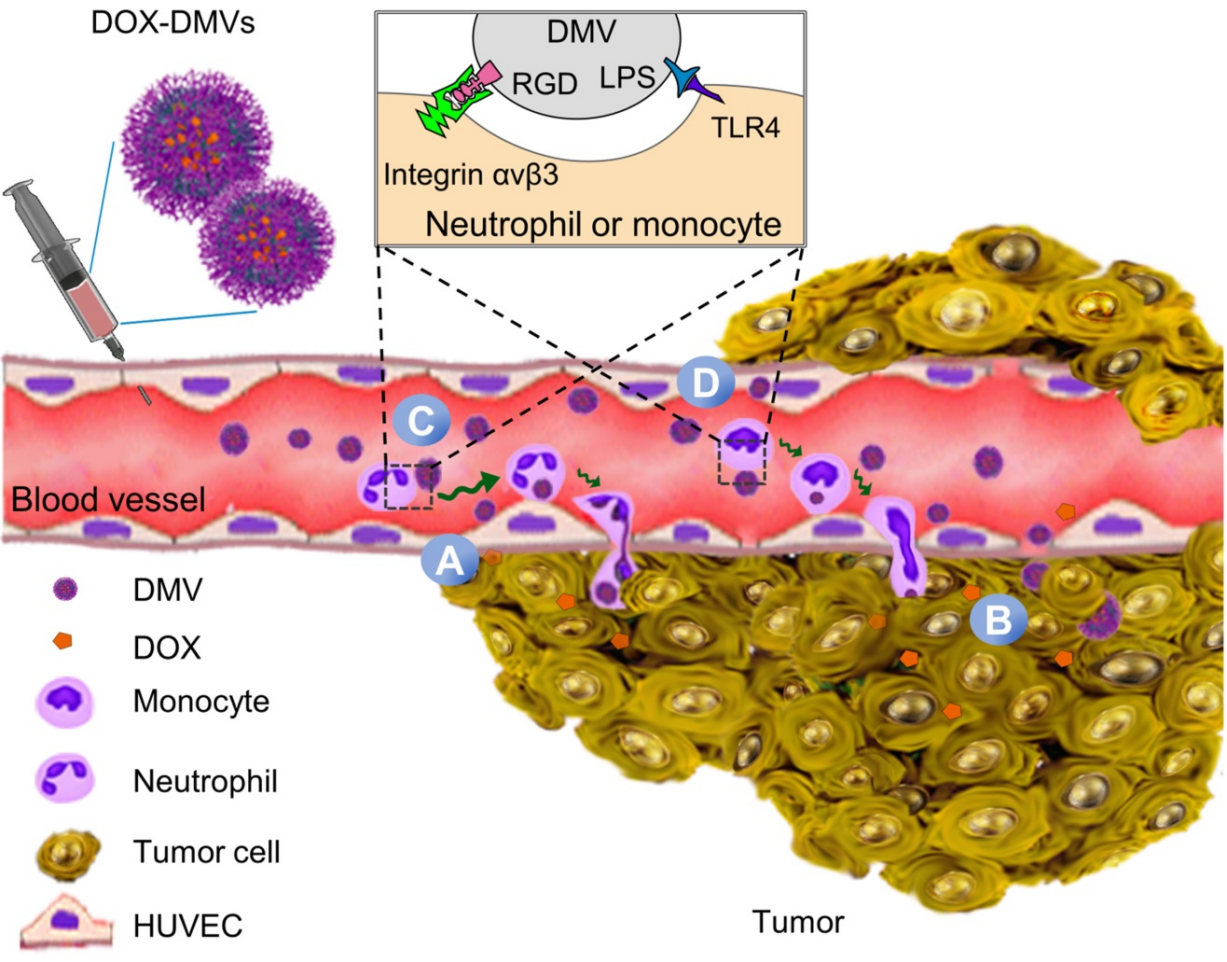
** Schematic of DOX-loaded RGD-EGFP-DMVs which target multiple cells to deliver DOX into tumor microenvironments.** DMVs bind to tumor vasculature (A) and tumor cells (B), generating the local inflammatory response to activate blood immune cells. Activated neutrophils and monocytes take up DMVs *via* interactions of RGD and LPS on DMVs with integrin α_v_β_3_ and TLR4 on neutrophils or monocytes, respectively. Transmigration of neutrophils (C) and monocytes (D) mediates the transport of DMVs across blood vessel barriers for their tumor accumulation. Targeting of DMVs to multiple cells in TMEs enhances delivery of DOX, thus increasing cancer therapy.

Here, we expressed a RGD peptide on the surface of DMVs to simultaneously target multiple cells (including neutrophils, monocytes, and tumor vasculature and tumor cells) for delivery of doxorubicin (DOX) into TMEs (Figure 1). In the studies, we utilized cytolysin A (ClyA) (34 kDa, pore-forming toxin 42, 43) to display a RGD4C peptide 17 on the surface of non-pathogenic *E. coli* BL21. Using nitrogen cavitation, we generated DMVs from *E. coli* BL21 expressing ClyA-RGD4C-EGFP. We developed a new method to remotely load DOX inside DMVs *via* the pH gradient-mediated drug loading. Our data showed that DOX-loaded RGD-EGFP-DMVs (DOX-RGD-EGFP-DMVs) can simultaneously target tumor vasculature and immune cells to improve the delivery of DOX into TMEs. Collectively, our study offers a new strategy to develop bacterium membrane-based nanovesicles that possess endogenous multiple cellular targeting features and the high drug loading for enhanced cancer therapies.

## Materials and Methods

### Chemicals and reagents

A pThois HisA ClyA-EGFP plasmid was given by Yanbing Ma's lab from Kunming in China. ClyA (Accession number: AF240780) has been proved to successfully express on the surface of *E. coli* 43. Endonucleases and polymerase chain reaction (PCR) reagents were purchased from New England Biolabs Inc. A bacterial strain of *E. coli* DH5α, BL21 was obtained from Thermo Fisher Scientific and was cultured in a Luria-Bertani medium (Thermo Fisher Scientific). The cell line of HL60 was bought from the American Type Culture Collection Center (ATCC) and the cell line of U937 was obtained from the researchers at the Washington State University. HL60 and U937 cells were cultured in RPMI1640 medium (R&D Systems) supplemented with 10% (v/v) fetal bovine serum (FBS) (Atlanta Biologicals) and penicillin/streptomycin (at 1% (v/v)) (Gibco). Human umbilical vein endothelial cells (HUVECs) (Lonza, Walkersville, MD) were cultured in a flask or a plate coated with 0.1% gelatin in an EBM medium supplemented with a kit comprised of FBS, rhEGF, hydrocortisone, GA-100, bovine brain extract and ascorbic acid (Lonza, Walkersville, MD) for 30 min. Melanoma B16-F10 cells (ATCC, Manassas, VA) were cultured in DMEM supplemented with 10% (v/v) fetal bovine serum, streptomycin (100 μg/mL) and penicillin (100 U/mL). Cells were incubated in a humidified incubator at 37 °C with 5% CO_2_ (Forma Steri-Cycle, Thermo Fisher Scientific). Dimethyl sulfoxide (DMSO), isopropyl-β-D-thiogalactoside (IPTG), cholesterol, collagenase type IV, PMA and LPS (lipopolysaccharide) antibody were obtained from MilliporeSigma. A lipid dye of DiR (1,1′-dioctadecyl-3,3,3′,3′-tetramethylindotricarbocyanine iodide) and recombinant EGFP proteins were purchased from Life Technologies Inc. Monoclonal antibodies of integrin α_v_, integrin β_3_, intercellular adhesion molecule-1 (ICAM-1), vascular cell adhesion molecule-1 (VCAM-1), glyceraldehyde 3-phosphate dehydrogenase (GAPDH) and TLR4 were obtained from Santa Cruz Biotechnologies. Proteins including N-formyl methionine-leucyl-phenylalanine (fMLP), IL-6, IL-1β and TNF-α, antibodies, including anti-EGFP, Alexa Fluor 488-anti-EGFP, Alexa Fluor 647-anti-CD31, Alexa Fluor 647-anti-LY-6G and Alexa Fluor 647-anti-F4/80 were bought from Biolegend. RGD peptides were purchased from Enzo life Sciences. The Cell Titer Aqueous One Solution Cell Proliferation Kit was obtained from Promega. Doxorubicin hydrochloride was purchased from Wuhan Yuancheng Gongchuang Technology Co. Ltd.

### Plasmid construction for expressing RGD4C-EGFP fusion protein

RGD4C and EGFP (Accession number: NC_011521.1) were synthesized from Thermo Fisher Scientific. RGD4C-EGFP was further sub-cloned into the pThois HisA-ClyA-EGFP plasmid using endonucleases BamHI and SalI. The primers of ClyA-Forward (5'-AAGGCACGTCATCTGACGTGCCT-3') and EGFP-Reverse (5'-ATTAAGTTGAACGCCAGG-3') were used for the subclone. The inserted sequence was verified by a full DNA sequencing (Genewiz, South Plainfield, NJ).

### Production of DMVs and their characterization

Engineered RGD4C-EGFP *E. coli* BL21 (1 L) were cultured at 0.5 mM of IPTG for 4 h, and then the bacteria were harvested followed by washing with HBSS (without Ca^2+^, Mg^2+^ and Phenol red, Corning, Inc, NY). The bacteria were collected and were re-suspended in HBSS at concentrations of 1-1.5 × 10^9^ /mL. The cell suspension (10-20 mL) was placed in a nitrogen cavitation vessel (Parr instrument, Moline, IL). The cells were under a pressure at 1500 psi for 20 min, the pressure was quickly released to disrupt cells. This procedure was repeated twice. The resulting suspension was centrifuged at 6 000 g at 4 ℃ for 30 min. The resulting supernatant was centrifuged at 100 000 g for 30 min at 4 ℃ (Ultra TLX, Beckman coulter, Brea, CA). The pellets were washed once with HBSS. After centrifugation, the supernatant was removed; EGFP-DMVs or RGD-EGFP-DMVs in pellets were suspended in HBSS (1 mL). DMVs from *E. coli* BL21 were served as control.

DMVs were characterized using dynamic light scattering (DLS) and cryo-TEM. The particle sizes and zeta potentials were measured by Malvern Zetasizer Nano ZS90 (Westborough, MA). Results were usually repeated five times. For cryo-TEM, a drop of the solution of DMVs (1 mg/mL) was deposited on a carbon-coated grid discharged by PELCO EasiGlow. After soaked by a piece of filter paper, the grid was quickly dropped in liquid nitrogen and stored overnight. The samples were imaged using TF20 TEM with a liquid nitrogen stage. The stability of DMVs was characterized by measurement of nanoparticle sizes using DLS over 7 days.

Protein profiles of *E coli*, RGD-EGFP-*E coli* and RGD-EGFP-DMVs were analyzed by electrophoresis on a 15% SDS-PAGE gel and each sample was loaded at 20 μg of proteins, followed by staining with Coomassie brilliant blue 250 for imaging. To identify the fusion protein ClyA-RGD4C-EGFP, separate proteins of each sample were transferred to polyvinylidene fluoride membrane and blotted with the monoclonal antibody for EGFP and polyclonal antibody for LPS.

### Identification of RGD4C-EGFP fusion protein on DMVs

Expression of a RGD4C-EGFP-ClyA fusion protein on DMVs was detected by immunoblotting. Briefly, samples were separated by SDS-PAGE and then transferred to a PVDF membrane, and RGD4C-EGFP was determined by anti-EGFP antibody. To confirm the RGD4C-EGFP fusion protein located on the surface, DMVs derived from *E. coli* or *E. coli* expressing pThois-HisA-ClyA-RGD-EGFP were treated with proteinase K at 0.1 µg/mL (PK, NEB) for 1 h at 37 ℃ to degrade surface proteins 44, and then the samples were analyzed by immunoblotting using anti-EGFP antibody. Furthermore, bacteria and DMVs were analyzed using flow cytometry to detect the fluorescence signal of EGFP.

### Cell markers detected by Western blots

The cells (such as non-differentiated or differentiated HL60, non-differentiated or differentiated U937, non-activated or activated HUVECs and B16-F10) were seeded at 10^5^ cells/well in a 12-well plate one day prior to the assay. After different treatments, the cells were washed and lysed using 0.1 mL of cell lysis buffer (Thermo Scientific, Rockford, IL) and were loaded on 12% SDS-PAGE for electrophoresis. Proteins were blotted using anti-integrin α_v_, anti-integrin β_3_, anti-ICAM-1, anti-VCAM-1 and anti-TLR4 monoclonal antibody. GAPDH was detected using anti-GAPDH antibody as the internal reference. The gray intensities of Western blots were obtained by the software of Image Lab (Bio-Rad, Hercules, CA).

### Simulation of ClyA-RGD4C-EGFP conformations

The molecular structure was simulated using the online software, Phyre2 45. The amino acid sequence of the fusion protein ClyA-RGD4C-EGFP was uploaded in Phyre2 to simulate protein conformations.

### Cellular uptake of DMVs visualized using confocal microscopy

For the adherent cells (HUVECs and B16-F10), 2 x 10^5^ cells were seeded in a dish (3.5 cm diameter) with a glass coverslip and cultured in a cell incubator overnight. HUVECs treated with TNF-*α* at 50 ng/mL for 4 h, or B16-F10 cells were incubated with DMVs, EGFP-DMVs or RGD-EGFP-DMVs at 37 ℃ for 60 min respectively, followed by fixing the cells with 4% paraformaldehyde (PFA) for 30 min. To determine the specificity of DMVs in binding to RGD, the cells were pretreated with free RGD peptides at 10 μM, and then RGD-EGFP-DMVs were incubated for 30 min. The equal fluorescence intensity of EGFP-DMVs and RGD-EGFP-DMVs was used for quantitative analysis of their cellular uptake. The samples were imaged using a confocal microscope (A1R plus, Nikon Japan).

HL60 cells and U937 cells are model cell lines of neutrophils and monocytes, respectively after their differentiation. To differentiate HL60 cells, DMSO (1.25% (v/v) in media) was added into HL60 cells and the cells were further cultured for 4 days. For differentiation of U937 cells, 10 ng/mL of PMA was added and the cells were cultured for up to 24 h. For uptake of non-adherent cells (differentiated HL60 and U937), 2 x 10^5^ cells were incubated with DMVs, EGFP-DMVs or RGD-EGFP-DMVs at 37 ℃ for 60 min respectively, followed by spreading of cells on a slide centrifuged at 800 g for 10 min and then the cells were fixed using 4% PFA for 30 min. To determine the specificity of DMVs in binding to RGD, the cells were pretreated with free RGD peptides at 10 μM, and then RGD-EGFP-DMVs were incubated with the cells for 30 min. The slides were mounted with a mounting media containing 4',6'-diamidino-2-phenylindole (DAPI) (BioVision, Milpitas, CA) and images were taken using a confocal microscope (A1R plus, Nikon, Japan).

### Quantitative analysis of cellular uptake of DMVs using flow cytometry

 Differentiated HL60 cells and U937 cells, HUVECs and B16-F10 melanoma cells were used in the studies. To activate HUVECs, TNF-*α* (50 ng/mL) was added into the medium 28, 46, 47. The cells were cultured in a flat-bottom 6-well plate at 2 × 10^5^ cells/well for 24 h, and the cells were then incubated with DMVs, EGFP-DMVs, or RGD-EGFP-DMVs at 12 µg/mL for 30 min at 37 ℃. To determine the specificity of DMVs in binding to RGD, the cells were pretreated with free RGD peptides at 10 μM, and then RGD-EGFP-DMVs were incubated with the cells for 30 min. After the treatments, the cells (HUVECs, B16-F10 cells, HL60 and U937 cells) were washed with HBSS 3 times before flow cytometry was performed. The uptake of DMVs was quantified by measurement of EGFP fluorescence using a Gallios flow cytometer (Gallios, Beckman coulter, Brea, CA).

### Tumor targeting of RGD-EGFP-DMVs in a melanoma mouse model

Adult wild type male mice of C57BL/6 (10-12 weeks) were purchased from Jackson Laboratory. The mice were maintained in a polyethylene cage with a stainless lid at 22 ℃ with a 12-h light/dark cycle and covered with a filter cap. Mice were fed with food and water ad lib. The Washington State University Institutional Animal Care and Use Committee has approved all animal care and experimental protocols used in the studies. All experiments were performed under anesthesia using intraperitoneal (*i. p.*) injection of a mixture of ketamine (120 mg/kg) and xylazine (6 mg/kg) in saline.

The tumor model was established by subcutaneously injecting 1 × 10^6^ of B16-F10 cells in 0.1 mL HBSS (pH 7.4) to a mouse hind flank. Tumors were measured every two days with a caliper. The volumes were calculated according to the following formula: volume = L × W^2^/ 2. Once the tumor reached a volume of 300-400 mm^3^, EGFP-DMVs or RGD-EGFP-DMVs (at 10 mg/kg) were *i.v.* administered to the mice. 3 h later, xenografted tumor tissues were isolated and embedded in optimal cutting temperature (OCT) compound for sectioning. The sections were fixed using 4% paraformaldehyde for 10 min. Primary antibodies, including rat monoclonal antibodies for LY-6G, CD31, and F4/80, were incubated with fixed tissues. After washed, the tissues were incubated with polyclonal goat anti-rat IgG antibodies. Alexa Fluor 488-anti-EGFP antibody was used to identify EGFP-DMVs and RGD-EGFP-DMVs.

Xenografted tumor tissues were cut into small pieces and digested with 60 U/mL of DNase I and 125 U/mL of collagenase type IV for 30 min at 37 ℃ 48. The single cell suspension was obtained through a 100-mesh filter and stained with Alexa Fluor 647-Ly-6G for neutrophils or PE-F4/80 (monocytes/macrophages). Flow cytometry was performed to detect the signals of EGFP, Alexa Fluor 647-Ly-6G or PE-F4/80 to determine the cellular uptake of DMVs.

### Adhesion and transmigration assays

In cell adhesion experiments, HUVECs were seeded on a 96-well plate at 10,000/well. 24 h later, the cells were activated with 150 μg/mL of EGFP-DMVs or RGD-EGFP-DMVs for 4 h. The differentiated HL60 and U937 cells were labeled with DiR and the DiR-labeled cells were added to each well at 10^5^ /well. The plate was incubated at 37 ℃ for 15 min. The cells in each well were washed once with PBS to remove non-bound HL60 and U937 cells, and then 0.2 mL of DMSO was added to extract DiR for quantification of cells adhered to HUVECs. The DiR signal was measured using the Synergy Neo fluorescence plate reader (BioTek, Winooski, VT).

The transmigration experiment was performed following the previously established method 29. Transwells made of a membrane (12 mm in diameter) with the pores (3 μm in diameter) were first coated with gelatin (0.2%) for 0.5 h at room temperature. HUVECs were added (10^5^ cells/well) to the transwells and cultured in a 24-well plate for 3 days until 100% of confluence. Cytokines and chemokines (10 ng/mL of fMLP, 10 ng/mL of TNF-α, 10 ng/mL of IL-1β and 10 ng/mL of IL-6) in 0.6 mL were added to the bottom well. 1 × 10^5^ of activated HL60 and U937 cells in 0.2 mL of cell culture medium along with 150 µg/mL of EGFP-DMVs or RGD-EGFP-DMVs were placed in the upper chamber. Transmigration was performed for 4 h at 37 °C. The cells were collected from the bottom chambers and gently washed with PBS buffer for cell counting.

### Biodistribution of RGD-EGFP-DMVs in a melanoma mouse model

The mouse tumor implantation was performed as described above. When the tumor size reached to 300-400 mm^3^, DiR-labeled RGD-EGFP-DMVs or EGFP-DMVs at 10 mg/kg (at same protein level of DMVs) were administrated intravenously *via* the tail vein. After 2 h or 24 h, tissues (heart, liver, spleen, lung, kidney) and tumors were excised. 100 mg of organ tissues or the tumor was minced in 1 mL HBSS and homogenized with a homogenizer of OMNI Tissue Master 125 (Kennesaw, GA) at 35,000 rpm in 1 min for three times at 4 ℃. The concentrations of DiR in tissues were measured by a Synergy Neo fluorescence plate reader (BioTek, Winooski, VT). Standards were prepared by diluting DiR stock solution (at 100 µg/mL).

### Toxicity of RGD-EGFP-DMVs

HUVECs, HL60 and U937 cells were seeded in a 96-well plate at a density of 10 000 cells/well and cultured overnight. RGD-EGFP-DMVs at various concentrations were incubated with the cells for 24 h at 37 ℃ and 10 μL of 3-(4,5-dimethylthiazol-2-yl)-5-(3-carboxymethoxyphenyl)-2-(4-sulfophenyl)-2H-tetrazolium (MTS) (Promega, Madison, WI) was added into each well. 4 h later, the absorbance at 490 nm was measured using a plate reader (BioTek, Winooski, VT). To study the *in vivo* toxicity of RGD-EGFP-DMVs in mice, the tumor-bearing mice were *i.v.* administered with DiR-labeled RGD-EGFP-DMVs (600 μg). The pharmacokinetics of RGD-EGFP-DMVs was determined by measuring DiR fluorescence intensities of plasma samples at predesignated time points within 24 h. The main organs, including heart, liver, spleen, lung and kidney, were collected at 24 h post-injection of DMVs and fixed by 10% formalin followed by sectioning for histology study. In addition, cytokines (TNF-α, IL-1β and IL-6) in plasma were determined by ELISA at 24 h post-injection.

### Remote loading of doxorubicin (DOX) inside DMVs

DOX was remotely loaded into engineered DMVs by a pH gradient. DMVs were made in ammonium sulfate at 0.3 M (pH 5.2) (Sigma-Aldrich, Saint Louis, MO) and the amount of DMVs was determined by a lyophilization assay. Cholesterol was dissolved in pyridine at 500 mg/mL. 10% of cholesterol over DMVs was added into the suspension of DMVs at 0.3 M of ammonium sulfate, followed by gentle mixing at 37 ℃ for 30 min. Subsequently, the ammonium sulfate buffer was replaced by saline or 5% glucose solution using centrifugation with a 100 kDa centrifugal filter (Millipore, Burlington, MA) at 6 000 g for 30 min. This process was repeated two more times. DMVs were mixed with doxorubicin hydrochloride solution. The mixture was incubated for 2 h at 37 ℃ and non-encapsulated DOX was removed by centrifugation through a filter (cutoff at 100 kDa). The drug loading in DMVs was quantified by measuring the absorbance of DOX at 480 nm using a microplate reader. The loading efficiency was calculated as the mass of the loaded drug divided by the mass of DMVs.

Loading efficiency (%) = 
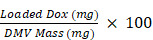


To optimize drug loading, the effects of ammonium sulfate, cholesterol and DOX inputs on drug loading efficiencies were studied. Drug release from DMVs was evaluated by loading 500 µl of DOX-DMVs into a dialysis unit with the cutoff at 100 kDa (Spectrum Inc.), followed by immersion into 20 mL PBS at pH 6.0 and 7.4. The samples were agitated at 90 rpm in a shaking incubator at 37 ℃. At predetermined time intervals, the samples were withdrawn from the outer media and an equal volume of fresh PBS was immediately replenished. The amount of drug release was determined by absorbance of DOX using a microplate reader. The size and zeta-potential of DOX-EGFP-DMVs were also measured using Malvern Zetasizer Nano ZS90 (Westborough, MA).

### Toxicity of DOX-EGFP-DMVs and DOX-RGD-EGFP-DMVs

B16-F10 cells were seeded in a 96-well plate at a density of 5 000 cells/well and cultured overnight. Free DOX, DOX-RGD-EGFP-DMVs or DOX-RGD-EGFP-DMVs at varying drug concentrations were then incubated with the cells for 24 h at 37 ℃. An MTS cell proliferation kit (Promega, Madison, WI) was used to assess cell viability according to the manufacturer instructions.

### Cancer therapy of DOX-RGD-EGFP-DMVs in the melanoma mouse model

The tumor model was established as described above. The anti-tumor efficacy was assessed 49. Once the tumor reached the volumes of 50-100 mm^3^, the mice were randomly separated into 6 groups including saline, free DOX (2 mg/kg in saline), EGFP-DMVs (0.75 mg/mouse), RGD-EGFP-DMVs (0.75 mg/mouse), DOX-EGFP-DMVs (2 mg/kg of DOX in 0.75 mg of DMVs) and DOX-RGD-EGFP-DMVs (2 mg/kg of DOX in 075 mg of DMVs). They were intravenously administered intravenously. 24 h later, the second dose was given to the mice. Tumor sizes and body weights were recorded daily. 10 days after therapy, xenografted tumor tissues were isolated and imaged.

### Statistical Analysis

Data are expressed as mean ± SD. Statistical analysis was conducted using one-way T-test using Origin 8.5. P values < 0.05 are considered significant (*). P values < 0.01 are considered very significant (**). P values < 0.001 are considered extremely significant (***).

## Results

### Physicochemical properties of RGD-EGFP-DMVs derived from non-pathogenic *E. coli*

**Figure 2:**
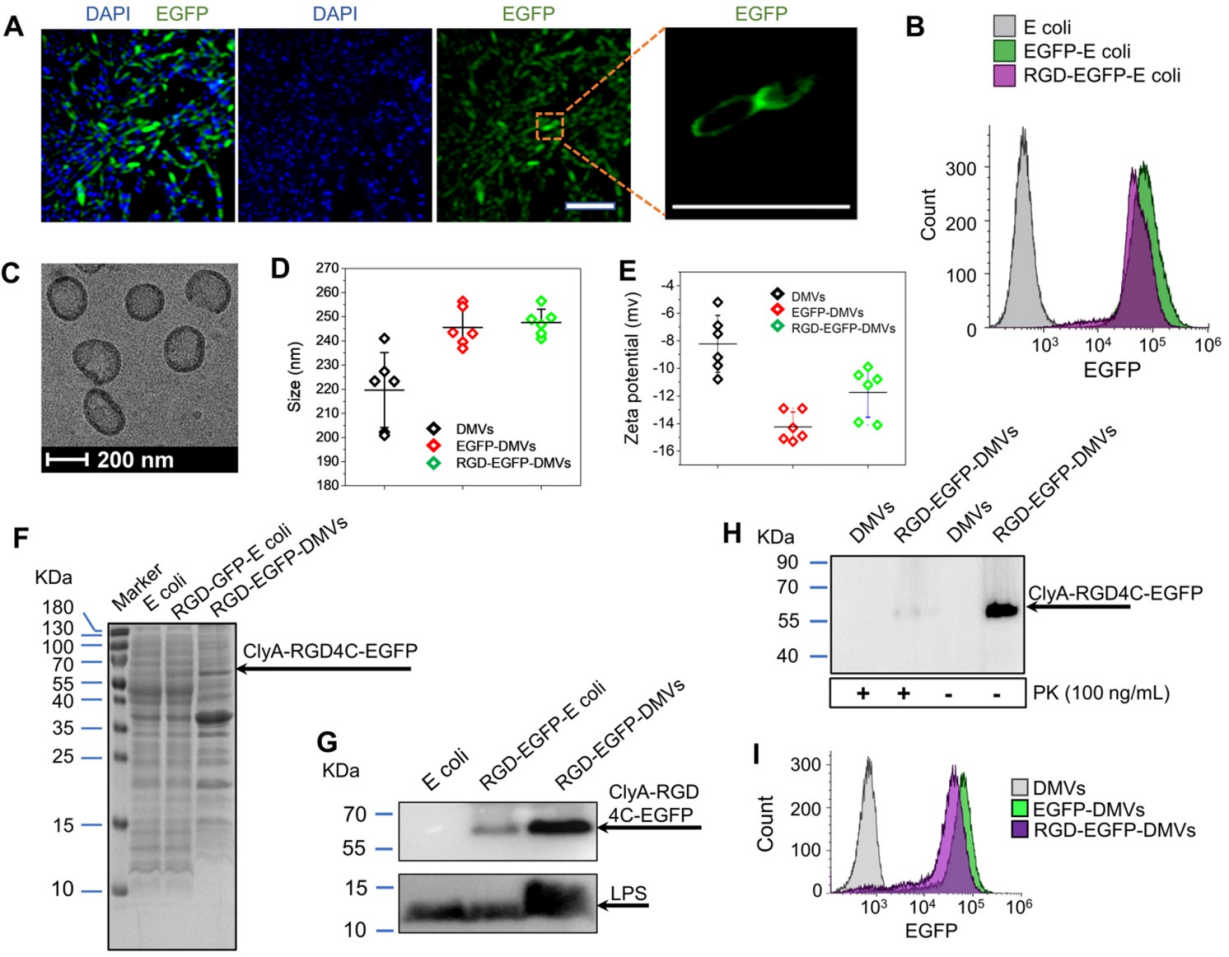
** Engineering of RGD peptides on the surface of *E. coli* and production of RGD-EGFP-DMVs. (A)** Confocal images of *E. coli BL21* expressing RGD-EGFP with a plasmid of pThioHisA-ClyA-RGD-EGFP. The enlarged image shows EGFP fluorescence of individual bacteria. Scale bar = 5 μm. **(B)** Flow cytometry of *E. coli*, EGFP-*E. coli*, and RGD-EGFP-*E. coli* by measuring EGFP signal. **(C)** Cryo-TEM image of RGD-EGFP-DMVs. Sizes **(D)** and zeta potentials **(E)** of DMVs, EGFP-DMVs and RGD-EGFP-DMVs determined by dynamic light scattering. Data were expressed as mean±SD (n = 5 - 6). **(F)** Protein profiles of *E coli*, EGFP-*E coli* and RGD-EGFP-DMVs. Each sample at 20 μg of proteins was loaded on SDS-PAGE. (G) Immunoblotting of ClyA-RGD4C-EGFP fusion protein expressed on RGD-*E. coli* and RGD-EGFP-DMVs using anti-EGFP monoclonal antibody to mark ClyA-RGD4C-EGFP protein. **(H)** Immunoblotting of RGD-EGFP on the surface of *E. coli* and RGD-EGFP-DMVs, with or without PK treatment (1 h at 100 ng /mL of PK), and the detection was using anti-EGFP antibodies. **(I)** Flow cytometry of DMVs, EGFP-DMVs and RGD-EGFP-DMVs.

Figure S1A shows the chemical structure and amino acid sequences of RGD4C. The RGD4C-EGFP was expressed on *E. coli* BL21. Figure S1B shows the DNA sequence of RGD4C highlighted in yellow and RGD4C was sub-cloned into a pThioHisA-ClyA plasmid (Figure S1C). The previous work has proved that ClyA can be used as an outer membrane protein linker for expression of Omp22 on the cell surface 43. In the present study, we expressed RGD4C at the C-terminus of ClyA on the surface of cells. EGFP was fused as a fluorescent tag at the C-terminus of RGD4C peptide for studying tissue targeting of DMVs *in vitro* and *in vivo*. The coding sequences in both pThioHisA-ClyA-EGFP and pThioHisA-ClyA-RGD-EGFP plasmids were verified by DNA sequencing. The recombinant *E. coli* BL21RGD4C bacteria were visualized under a confocal microscope as shown in Figure 2A, revealing the EGFP tag on bacterial membrane. Furthermore, we measured EGFP signals of bacteria using flow cytometry, and found that both EGFP-*E. coli* and RGD-EGFP-*E. coli* possessed EGFP tags (Figure 2B). We also observed that adding of RGD decreased the EGFP fluorescence signal which may be due to the post-translation efficiency of proteins.

Bacteria were disrupted by nitrogen cavitation force, and subsequently the cytosol was released and bacterial double-layer membrane nanovesicles (DMVs) were formed 35. Using the nitrogen cavitation approach 35, we produced DMVs from *E. coli* and bioengineered *E. coli*. To study the intact structure of DMVs, we exploited cryogenic transmission electron microscopy (cryo-TEM) to image DMVs. Bioengineered RGD-EGFP-DMVs demonstrated spherical membrane structures with the size of around 200 nm in diameter (Figure 2C). The wall thickness of DMVs is similar to that of their parent bacteria 35. This structure of DMVs is different from OMVs because DMVs possessed the double-layer membrane of their parent bacteria. Furthermore, dynamic light scattering (DLS) measurement showed that the average size of RGD-EGFP-DMVs was 247 ± 5.5 nm, which was similar to that of EGFP-DMVs (245 ± 8.0 nm) (Figure 2D). We also measured the zeta potentials of DMVs, and the results (Figure 2E) showed that RGD-EGFP-DMVs and EGFP-DMVs appeared negative charges and they were similar, suggesting that both DMVs had the similar surface property. It was noted that the introduction of EGFP or RGD-EGFP to DMVs increased their sizes and decreased surface zeta potentials compared to native DMVs. This suggests that the protein expression of EFGP or RGD-EGFP may be outside bacterial membrane, thus affecting the surface properties of DMVs.

We also analyzed the protein profiles of *E. coli*, RGD-EGFP-*E. coli* and RGD-EGFP-DMVs using SDS-PAGE (Figure 2F). Their protein patterns were similar, indicating that RGD-EGFP-DMVs contained proteins of their parent bacteria. However, some proteins below 15 kDa were decreased in DMVs compared to their parent bacteria. These proteins may be associated with cytoplasmic components of bacteria because production of DMVs removed cytoplasmic proteins 35. We also observed that some proteins were increased in DMVs, for example, the protein at 65 kDa that may be associated with the protein, ClyA-RGD-EGFP. The immunoblotting of RGD-EGFP-DMVs and their parent bacteria (Figure 2G) supported our idea. We observed in Figure 2G that bioengineered *E. coli* BL21 RGD4C-EGFP appeared a band at 65 kDa that was responsible for ClyA-RGD-EGFP. Furthermore, we observed that DMVs contained LPS and RGD-EGFP compared to their parent RGD-EGFP *E. coli*, indicating that DMVs maintained tissue targeting of their parent bacteria. It is interesting to observe that LPS and RGD-EGFP were significantly increased in DMVs compared to those in their parent cells. This may be due to the production of DMVs comprised of only bacterial membrane 35.

To further confirm the RGD4C-EGFP fusion protein was present on the surface of RGD-EGFP-DMVs, proteinase K (PK) was used as a digest agent. The result (Figure 2H) showed that the ClyA-RGD4C-EGFP fusion protein at 65 kDa was observed, but it disappeared when PK was added. The results indicated that DMVs contained the RGD-EGFP component on their surface. To further confirm the EGFP tag on DMVs, we analyzed the DMVs using flow cytometry. The results showed that DMVs contained EGFP (Figure 2I). To estimate the number of RGD-EGFP on DMVs, we performed the Western blot (WB) experiments of EGFP and RGD-EGFP-DMVs (Figure S2). We compared the intensity of EGFP to that of RGD-EGFP-DMVs, estimating that there were around 200 EGFP molecules per DMVs. Furthermore, we measured the stability of DMVs using DLS. The results in Figure S3 showed that DMVs did not change their sizes in 7 days stored at 4 ℃. Collectively, the results show that DMVs may be excellent drug carriers for targeted drug delivery.

### RGD-EGFP-DMVs interact with multiple cells

**Figure 3:**
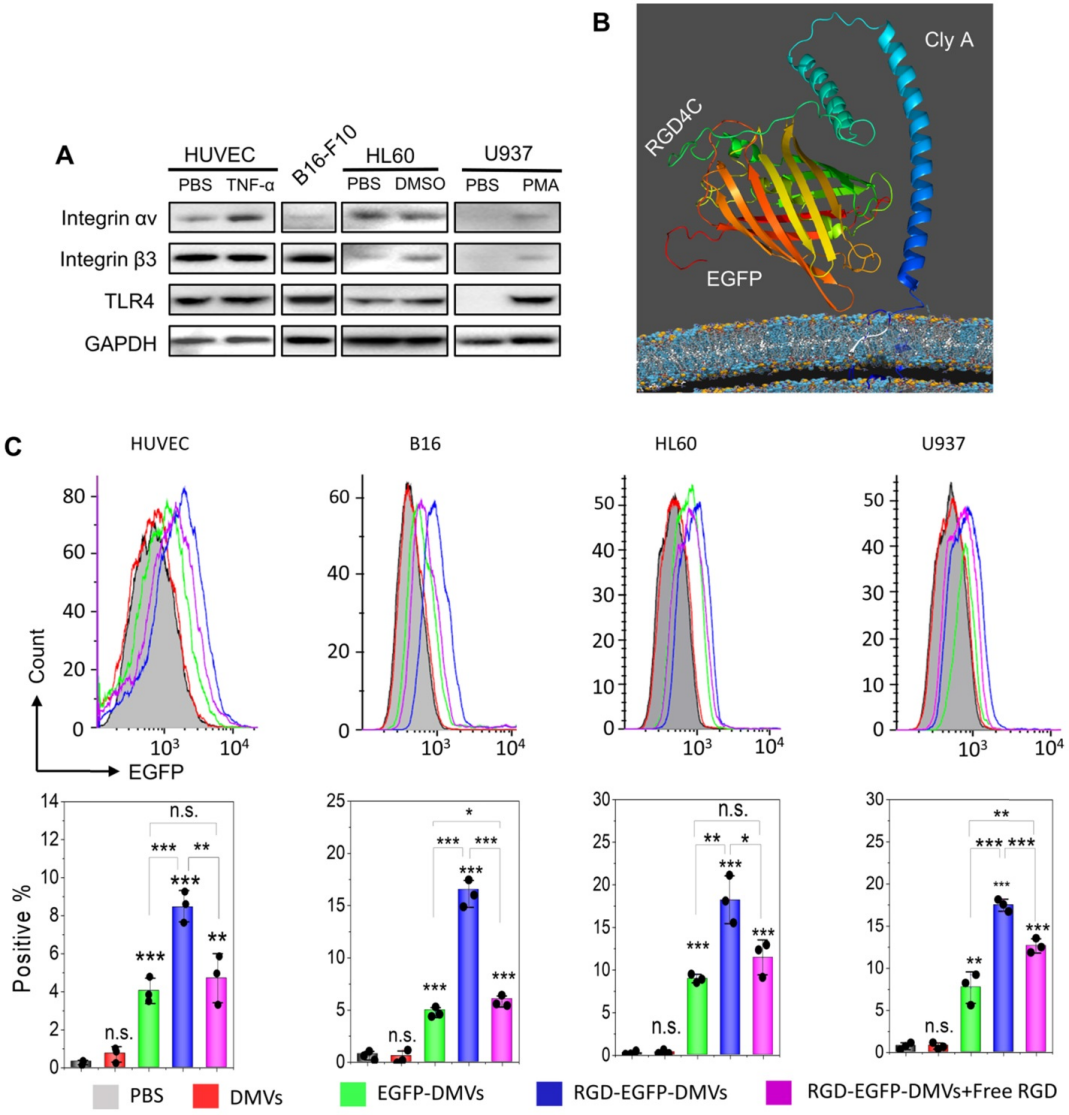
** RGD expressed on DMVs is required for their interactions with multiple cells *in vitro*. (A)** Western blots of integrin α_v_β_3_ and TLR4 on HUVECs, B16-F10 (melanoma cells), HL60 cells (neutrophils), and U937 cells (monocytes). **(B)** Conformation of the fusion protein ClyA-RGD-EGFP on the surface of bacteria simulated using Phyre2 software.** (C)** Flow cytometry (top) and its quantification (bottom) of cellular uptake of EGFP-DMVs and RGD-EGFP-DMVS by HUVECs, B16-F10, differentiated HL60 and differentiated U937 cells after the cells were incubated with DMVs at 12 µg/mL for 60 min. Data are represented as means ± SD, n = 3. *P < 0.05, * * *P <* 0.01, * * * *P <* 0.001.

Integrin α_v_β_3_ is the key receptor of RGD peptides. TMEs are inflammatory environments 39, 50. Endothelial vessels, tumor cells and immune cells may regulate the expression of integrin α_v_β_3_. TNF-*α* is known to activate endothelial cells, promoting the expression of integrin α_v_β_3_ on the surface 46. Lipopolysaccharide (LPS) is expressed on bacterial surface, and its receptor, Toll-like receptor 4 (TLR 4) expressed on immune cells, such as neutrophils 51 and monocytes 52. We addressed whether these cells expressed integrin α_v_β_3_ *in vitro*. In Figure 3A, we observed that integrin α_v_ in HUVECs was upregulated after TNF-*α* treatment. Tumor cells (B16-F10) expressed integrin β_3_. HL60 cells are neutrophil model cell lines 53, and DMSO can initiate their differentiation to neutrophils. We observed the expression of integrin α_v_β_3_ in differentiated HL60 cells. Similarly, we observed that monocytes (U937) 54 also expressed integrin α_v_β_3_ after their differentiation by phorbol 12-myristate 13-acetate (PMA). We also studied the expression of TLR4 on endothelial cells, B16-F10, HL60 cells and U937 cells, and found that TLR4 existed or was upregulated after their activation. DMVs possessed the RGD peptide and LPS as shown in Figure 2G, thus they may interact with those cells.

Next, we addressed the conformation of ClyA-RGD-EGFP on the bacterial surface by molecular simulation using Phyre2 and found that the RGD domain was exposed outside EGFP protein, thus the EGFP protein linked to DMVs would not prevent the binding of RGD to integrin α_v_β_3_ on targeted cells (Figure 3B). To prove our hypothesis, we performed the uptake of DMVs in several cell types (such as, activated endothelial cells, B16-F10, differentiated HL60 and differentiated U937). Using confocal fluorescence microscopy (Figure S4), we found that cellular uptake of DMVs was dependent on RGD expressed on DMVs compared to several controls (such as, DMVs, and EGFP-DMVs). When the cells were incubated with free RGD peptides, the uptake of RGD-EGFP-DMVs was significantly reduced. This shows that RGD expression on DMVs is required for their cellular uptake.

To quantitatively analyze the uptake of DMVs in the cells, we performed flow cytometry. Activated HUVECs, B16-F10 melanoma cells, HL60 cells or U937 cells were treated with RGD-EGFP-DMVs, EGFP-DMVs or DMVs at 6 µg/mL (DMVs were quantified by total proteins) for 1 h, respectively. Flow cytometry and their quantification (Figure 3C) showed that RGD-EGFP-DMVs were efficiently internalized compared to the controls (EGFP-DMVs and DMVs), suggesting that RGD was required for the cellular uptake of DMVs. To address the specificity of RGD in the uptake of DMVs, we pre-incubated free RGD peptides with cells, then we incubated them with DMVs after removing unbound RGD peptides. We performed flow cytometry to determine the uptake of DMVs. The results in Figure 3C showed that free RGD peptides inhibited the uptake of RGD-EGFP-DMVs by the cells (HUVEVs, B16 cells, HL60 cells and U937 cells). Collectively, the results indicate that RGD expressed on DMVs is required for their cellular uptake.

### Multiple cellular targeting of DMVs improves their accumulation in tumor sites

**Figure 4:**
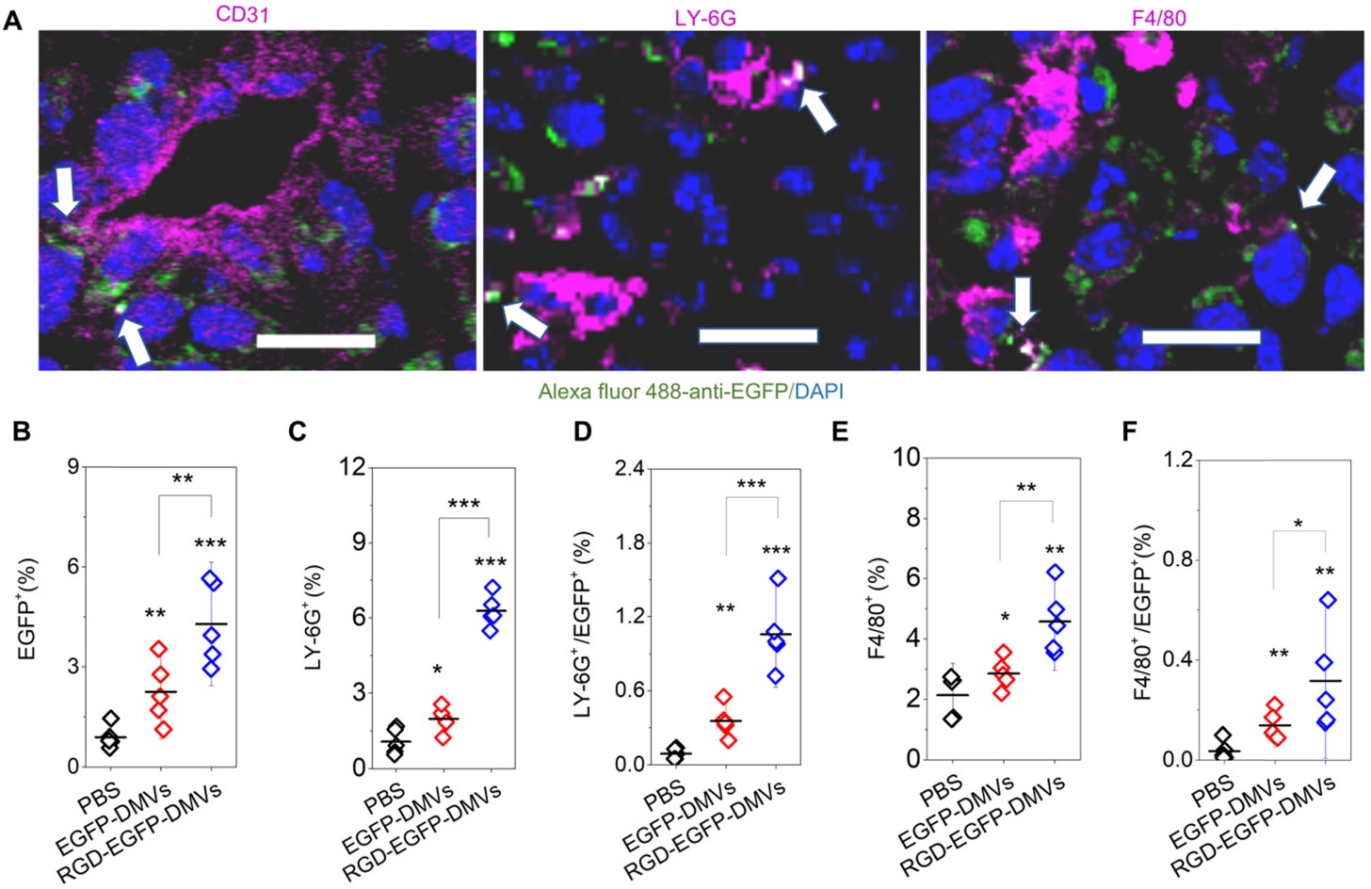
** RGD expression on DMVs is required for their cellular uptake by neutrophils and monocytes to mediate the transport of DMVs into tumor tissues. (A)** Colocalization of RGD-EGFP-DMVs with tumor vasculature stained by anti-CD31, neutrophils stained by anti-LY-6G and monocyte/macrophages stained by F4/80 antibody. Alexa 488-anti-EGFP antibody was used to identify RGD-EGFP-DMVs. Arrows indicate the colocalizations of cells and DMVs.** (B)** Flow cytometric analysis of total cells of tumor tissues for DMVs positive (n = 5). **(C and D)** Flow cytometric analysis on the uptake of DMVs by neutrophils (anti-LY-6G) and monocytes/macrophages (anti-F4/80) **(E and F)** in tumor tissues (n = 5). 3 h after injection of DMVs, the tumor tissues were digested with collagenase type IV to obtain single cell suspensions. The flow cytometry data are in Figure S5. Data were expressed as means ± SD. *P < 0.05, * * *P <* 0.01, * * * *P <* 0.001 compared to PBS group unless specified otherwise.

To examine how DMVs interacted with tumor microenvironments, we imaged tumor tissues after RGD-EGFP-DMVs were intravenously administered to mice implanted with a tumor. To locate endothelial cells, neutrophils and monocytes in tumor lesions, they were stained with their specific antibodies, respectively. As shown in Figure 4A, it was observed that DMVs interacted with endothelial cells labelled by anti-CD31, neutrophils stained with LY-6G and monocytes stained with F4/80, respectively. To quantitatively assess the interactions between DMVs and cells in TMEs, we obtained single cells from tumor tissues and performed flow cytometry to study the uptake of DMVs. First, we measured all cells of EGFP positive in tumor tissues (Figure 4B), and observed that the cells were increased in the case of administration of RGD-EGFP-DMVs to mice compared to EGFP-DMVs, suggesting that RGD peptides expressed on DMVs are required for their tumor accumulation (Figure 4B).

Next, we addressed what cell types mediated the transport of DMVs in tumor tissues. We studied the population of LY-6G positive cells which were neutrophils (Figure 4C and Figure S5). When DMVs were administered, neutrophils increased from 1% to 5.5%. There was the similar trend of increase for the cells positive for both LY-6G and DMVs (Figure 4D). In addition, we found that RGD-EGFP-DMVs were markedly increased in neutrophils (LY-6G positive cells) compared to EGFP-DMVs (Figure 4D). Similarly, we studied monocytes in tumor tissues and their uptake of DMVs (F4/80 positive cells). We observed that RGD-EGFP-DMVs increased an accumulation of monocytes in the tumor compared to EGFP-DMVs (Figure 4E). Furthermore, it was observed that RGD expression on DMVs enhanced the uptake of DMVs (Figure 4F). Collectively, the results indicate that RGD mediated the uptake of DMVs in neutrophils and monocytes, and subsequently the cells transported DMVs across tumor blood vessels.

**Figure 5:**
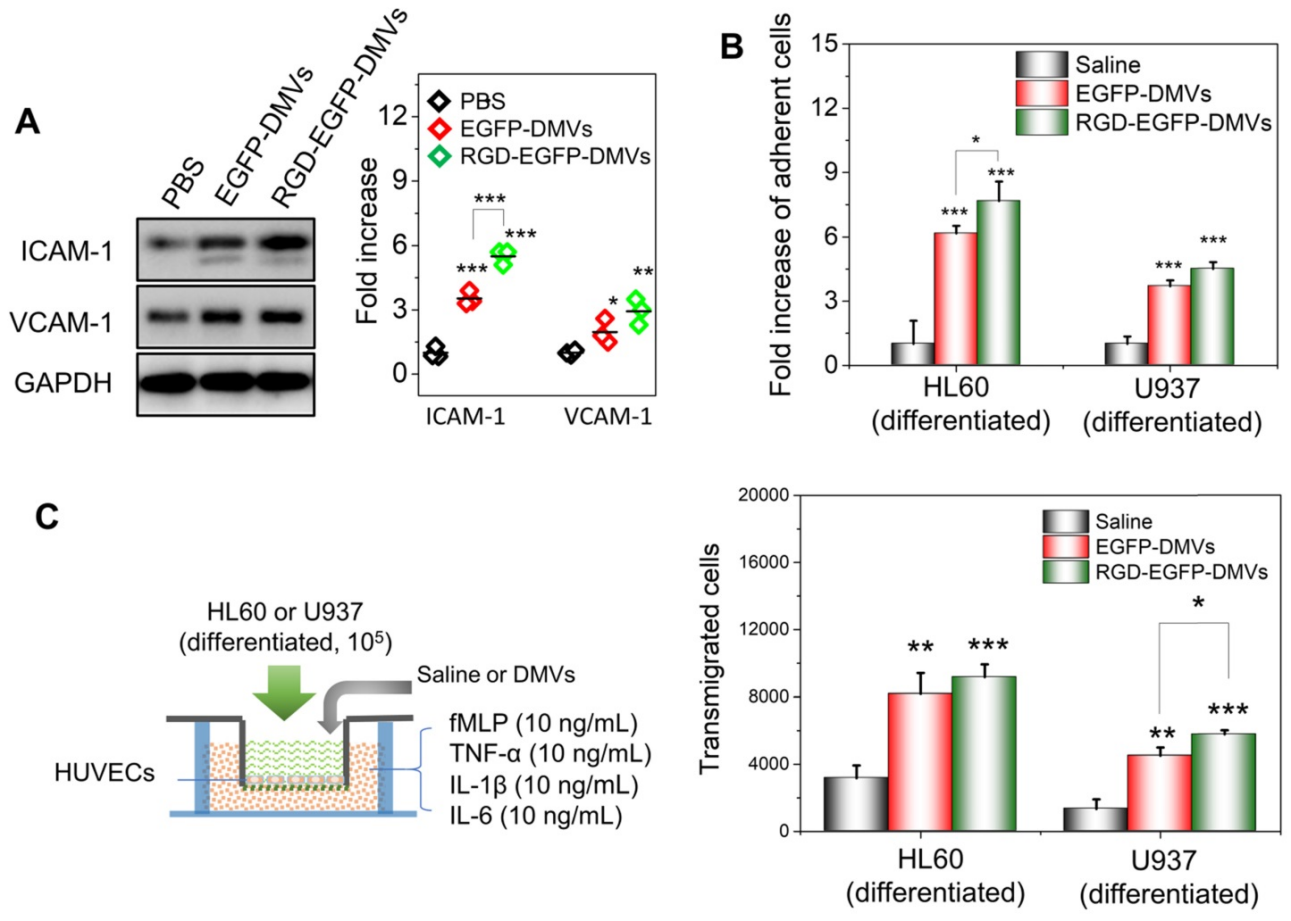
** DMVs activate endothelial cells to promote infiltration of neutrophils and monocytes across the endothelial barrier.** (**A**) Western blots of ICAM-1 and VCAM-1 on HUVEVs after the treatment with DMVs, and the quantification of Western blot results (right). (**B**) Adhesion of differentiated HL60 cells and U937 cells (stained by a lipid dye of DiR) to HUVECs treated by DMVs at 150 μg/mL for 4 h. (**C**) Transmigration of differentiated HL60 cells and U937 cells in the Transwell assay after HUVECs grew to a monolayer and the transmigration was performed in the presence of DMVs at150 μg/mL for 4 h. The scheme of experiments is illustrated on the left, and the results are shown on the right. Data were expressed as mean ± SD, n = 3 unless otherwise specified. *P < 0.05, * * *P <* 0.01, * * * *P <* 0.001 compared to PBS control group unless specified otherwise.

To address the molecular mechanism in which immune cells infiltrated in tumor tissues, we asked whether DMVs initiated the inflammatory response for cell transmigration when they interacted with endothelial cells. We incubated HUVECs with DMVs and found that DMVs upregulated the expression of ICAM-1 and VCAM-1 (Figure 5A). In addition, RGD-expressed DMVs showed the increased expression of ICAM-1 compared to non-targeted DMVs, suggesting that RGD-EGFP-DMVs may bind endothelial cells to initiate inflammatory responses due to LPS expression on DMVs. We also performed the binding of immune cells (differentiated HL60 cells and U937 cells) to endothelial cells and their transmigration using Transwell assays (Figure 5B and C) after endothelial cells were treated with DMVs. The results showed that indeed DMVs promoted immune cell adhesion and transmigration across endothelial cells. These processes are associated with DMVs that mediated endothelial inflammatory responses. The studies are consistent with enhanced tumor accumulation of neutrophils and monocytes that transported DMVs across tumor blood vessels (as shown in Figure 4).

We also investigated the pharmacokinetics of RGD-EGFP-DMVs in the tumor mouse model (Figure S6). We found that DMVs circulated for a long time (lasting 24 h) which assisted the binding of DMVs to tumor vasculature. Next, we studied the biodistribution of DMVs at 2 h or 24 h after EGFP-DMVs or RGD-EGFP-DMVs were intravenously administrated into melanoma bearing mice. The major organs and tumor tissues were dissected, and DMVs were detected by fluorescence (Figure S7). While we observed the accumulation of DMVs in the liver and spleen, we still observed the strong signals of DMVs in tumors, and their tumor accumulation increased with time. This is consistent with the long circulation times of DMVs (Figure S6). Most importantly, RGD-EGFP-DMVs were increased in the tumor tissues compared the control (without RGD), suggesting that RGD mediated the tumor deposition of DMVs. The results are consistent with the flow cytometry results as shown in Figure 4. We also tested the toxicity of DMVs on HUVECs, HL60 and U937 cell lines and no obvious cell death was observed (Figure S8).

Collectively, our results indicate that DMVs can trigger tumor endothelial inflammation response to activate neutrophils and monocytes in the circulation. RGD-DMVs were internalized by activated neutrophils and monocytes *via* the binding of RGD and LPS to integrin α_v_β_3_ and TLR4 respectively, therefore neutrophils and monocytes mediated the transport of DMVs across tumor blood vessels. DMVs can also target tumor vessels and tumor cells. The expression of RGD on DMVs mediated multiple cellular targeting to enhance the accumulation of DMVs in TMEs. DMVs did not show the toxicity to several cells and had the long circulation times. Therefore, DMVs are excellent drug carriers to deliver therapeutics in the tumor tissues.

### Efficient loading of DOX in DMVs *via* a pH gradient

**Figure 6:**
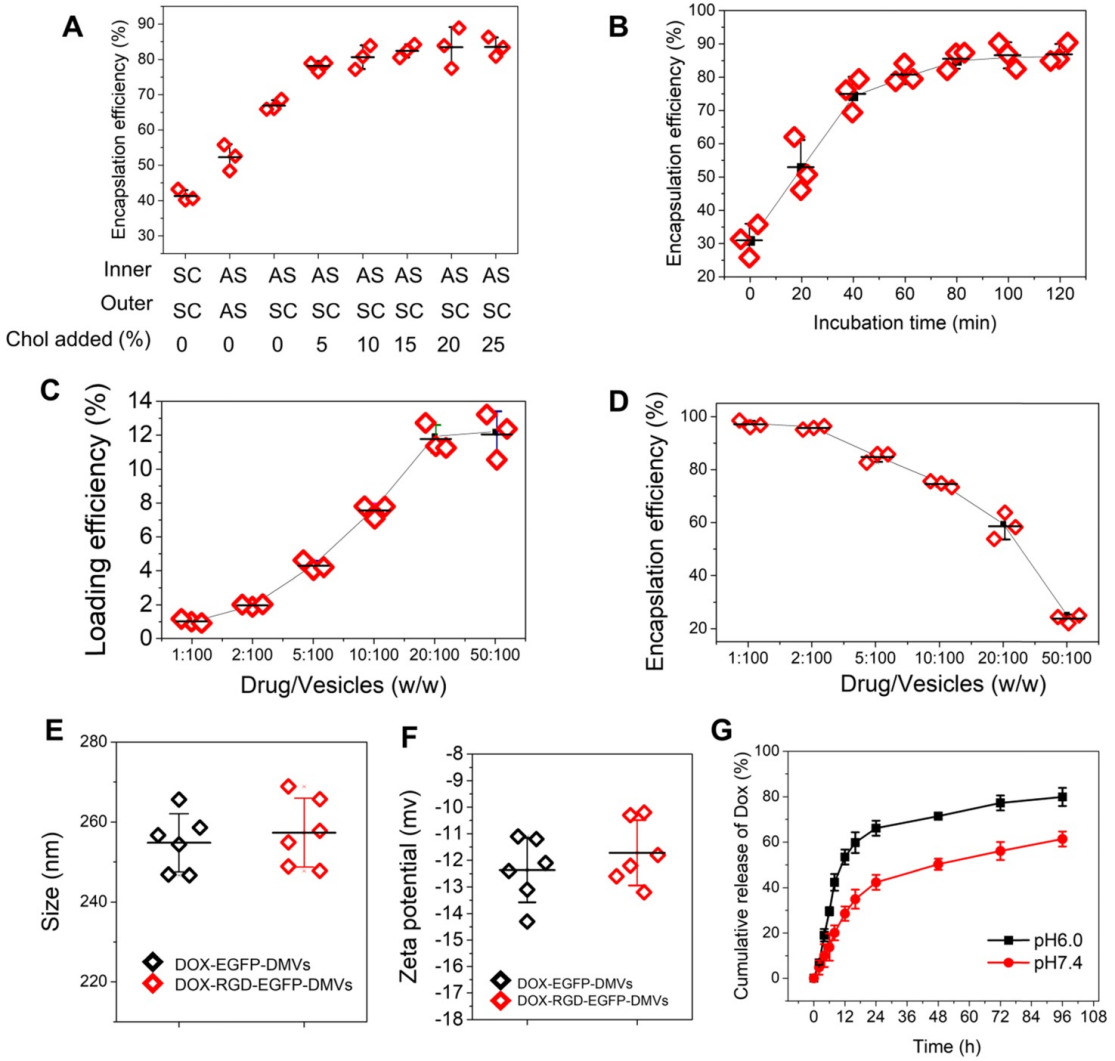
** High loading of DOX inside DMVs *via* pH gradient. (A)** Enhanced encapsulation of DOX by addition of cholesterol to the membrane of DMVs. The addition of cholesterol decreased membrane permeability to maintain the pH gradient between inner and outer membrane of DMVs. Loading DOX to RGD-EGFP-DMVs was prepared in ammonium sulfate at 300 mM (pH5.4) or saline. Cholesterol at 0-25% (w/w) into DMVs was added before DOX was added to the suspension of DMVs, and then drug encapsulation efficiencies were measured by a spectrometer. The ratio of drug over DMVs was at 2.5:100; Sodium chloride solution and ammonium sulfate solution denoted by SC and AS, respectively. Cholesterol denoted by Chol. **(B)** Incubation times for DOX loading efficiency. The experiments were conducted at 37 ℃ and the drug input at 2.5% to DMVs loaded with 10% Chol. The drug loading efficiency **(C)** and encapsulation efficiency **(D)** of DOX-DMVs when DMVs were set at 1 mg. Sizes **(E)** and zeta potentials **(F)** of DMVs after they were loaded with DOX. **(G)** DOX release profiles of DOX-RGD-EGFP-DMVs in PBS (pH7.4 or pH6.0) at 37 ℃. Data were expressed as mean ± SD, n = 3 unless otherwise specified.

The clinical liposome-based doxorubicin (so-called Doxil^®^) has been used to treat many cancers, and doxorubicin (DOX) is loaded inside liposomes *via* remote loading methods 55. Cholesterol is a major component of cell membrane and increases the integrity of phospholipid bilayer structure 56, thus maintaining a pH gradient between the inner and the outer liposomal membrane. This gradient can be exploited to load high concentrations of DOX inside liposomes. Figure 2C showed DMVs were liposomal structures, so it possible to load DOX inside DMVs. To decrease the membrane permeability of DMVs, we added cholesterol to DMVs after DMVs were produced in 300 mM ammonium sulfate solution. Then we exchanged the buffer to saline or 5% glucose solution to create a pH gradient between the inner and outer membrane of DMVs. DMVs in the HBSS buffer (pH7.2) were the control. When cholesterol was not added to DMVs in which there was no pH gradient (the inner and outer DMVs are in SC (sodium chloride) or AS (ammonium sulfate) buffer), we observed the low encapsulation efficiencies of DOX inside DMVs (Figure 6A). When we generated the pH gradient and added cholesterol to DMVs, we found that DOX encapsulation efficiencies in DMVs were dramatically increased and the efficiencies were saturated after 10% cholesterol was added (Figure 6A). This result indicated that the pH gradient and addition of cholesterol to DMVs were critical to increase DOX loading. We also studied the time course of DOX loading under the conditions of drug input (2.5%) over DMVs and 10% cholesterol (Figure 6B). We found that the incubation period of DOX and DMVs was critical to optimize the drug loading. Furthermore, we investigated the loading efficiency and encapsulation efficiency of DOX in DMVs when 10% cholesterol was added and the incubation was set for 2 h. With increasing the ratios between drug and DMVs, the loading efficiency was markedly increased, and it achieved to12% (w/w) of DOX over DMVs, and the encapsulation efficiency was 60% (as shown in Figure 6C and D).

We also measured the size and surface charges of DMVs after they were loaded with DOX (Figure 6E and F). The loading of DOX in RGD-expressed DMVs or non-RGD-expressed DMVs did not change their size and zeta potentials compared to those before the loading of DOX to DMVs (Figure 2D and E). The results indicated that loading of DOX did not affect the properties of native DMVs. The drug release was evaluated over 96 h at different pH values. The DOX release was sustained in a long period (more than a few days) and the acid environment (at pH 6.0) increased the release of DOX. The increased drug release may benefit cancer therapy because TMEs are acidic 57.

### DOX-RGD-EGFP-DMVs enhanced cancer therapy

**Figure 7:**
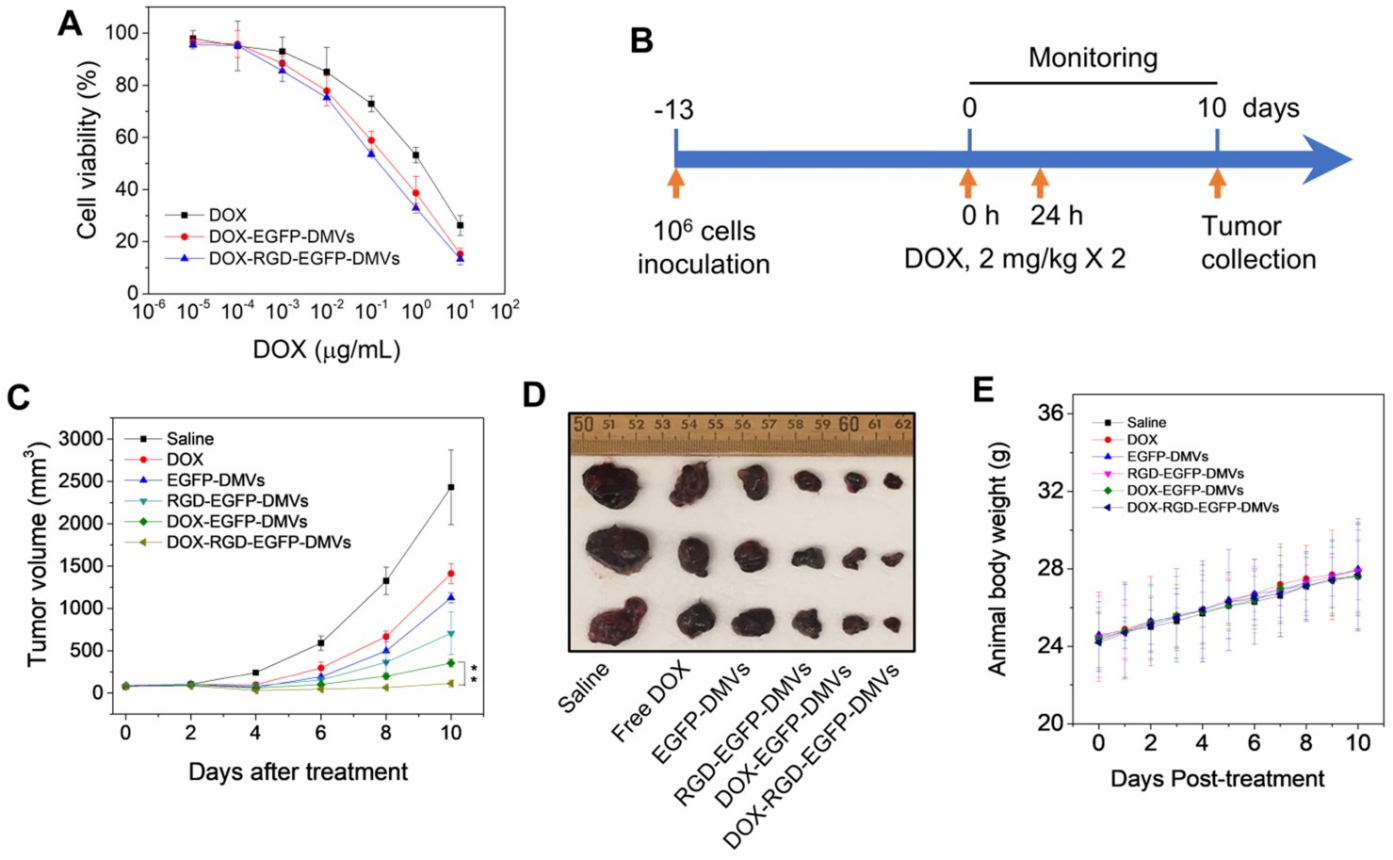
** RGD expressed on DMVs enhances cancer therapy. (A)** Cytotoxicity of free DOX, DOX-loaded RGD-EGFP-DMVs and DOX-loaded EGFP-DMVs to B16-F10 melanoma cells after the cells were treated for 24 h (n = 4). **(B)** Animal experimental design includes twice treatments of DOX formulations in cancer therapy. **(C)** Tumor growth after treatments with several formulations (n = 3 for PBS group; n = 6 for other groups). **(D)** Representative tumor images 10 days after the treatments with several formulations. **(E)** Body weights of animals during cancer therapy (n = 3 for the PBS group; n = 6 for the other groups). Data were expressed as mean ± SD. * * *P <* 0.01.

To evaluate the toxicity to tumor cells, free DOX or DOX-loaded DMVs were incubated with B16-F10 melanoma cells at various doses for 24 h. DOX-loaded DMVs showed the similar toxicity to free DOX, suggesting that DOX was released from DMVs to kill the tumor cells (Figure 7A).

We further examined the anti-tumor therapy of DOX-RGD-EGFP-DMVs in the melanoma mouse model and the experimental protocol was shown in Figure 7B. After the tumor volumes reached to 50-100 mm^3^, six groups of animals were established including HBSS, free DOX, EGFP-DMVs, RGD-EGFP-DMVs, DOX-EGFP-DMVs and DOX-RGD-EGFP-DMVs. We monitored the tumor growth for 10 days (Figure 7C) and found that tumor still grew after the treatments of free DOX, DMVs and DOX-loaded EGFP-DMVs. However, DOX-RGD-EGFP-DMVs markedly inhibited the tumor growth. We also imaged the real tumor size at Day 10 (Figure 7D), and the result was consistent with the tumor sizes as shown in Figure 7C. Interestingly, we observed that DMVs themselves decreased the tumor growth compared to free DOX. This may be associated with immune cell tumor infiltration to initiate cancer immunotherapy because we found that DMVs can cause inflammatory responses to mediate neutrophil and monocyte transmigration (Figure 4 and Figure 5). The mouse weights were monitored during the therapy and it was shown that our formulations of DMVs did not show the weight loss of animals compared to that of animals treated with saline (Figure 7E). This result is agreement with the *in vitro* cytotoxicity studies in which DMVs did not cause the cellular death in HUVECs, neutrophils and monocytes (Figure S8).

As we have shown that DMVs contained LPS from their parent bacteria, it is not clear whether DMVs caused systemic inflammation responses and whether this inflammation resulted in organ damage. To address these concerns, we measured the cytokines (such as TNF-*α*, IL-1β and IL-6) after we intravenously administered DMVs at 0.6 mg of DMVs which was the same dose used in the tumor therapy. It is noted that DMVs increased the levels of TNF-*α* and IL-6 (Figure S9). This inflammation response is needed to mediate the transport of neutrophils and monocytes to deliver DMVs in tumor tissues. To determine whether this temporal inflammation response caused tissue damage, we performed the histology of five major organs, such as heart, liver, spleen, lung and kidney (Figure S10). The histological results suggested that we did not observe the tissue damage in these organs, implying that the inflammation response may not lead to organ dysfunctions during cancer therapy.

## Discussion

Effectively treating cancers requires delivery systems to deliver drugs into tumor tissues, thus minimizing the toxicity 1, 58. TMEs are comprised of tumor cells, immune cells and blood vessels. Blood vessels are a biological barrier to prevent the permeability of drugs to tumor tissues. Ideal drug carriers can simultaneously target multiple cells to overcome the physiological barriers 20, 40, 49. To achieve these goals, we expressed RGD on the surface of bacteria (*E. coli* BL21) and added an EGFP link to maintain RGD function on the surface which also is an imaging probe to track DMVs *in vitro* and *in vivo*. Furthermore, bacteria possess endogenous ligands (such as LPS) that may increase tissue targeting of DMVs. *In vitro* and *in vivo* studies show that DMVs bind tumorous vasculature, mediating local inflammatory responses (such as ICAM-1 upregulation on endothelial cells) to recruit neutrophils and monocytes. During this process, administered DMVs can bind to activated neutrophils and monocytes *via* interactions of RGD and LPS on DMVs with integrin α_v_β_3_ and TLR4 on immune cells. Subsequently, these immune cells take up DMVs and transport them across the blood vessel barrier. Furthermore, DMVs can directly target tumor vasculature and tumor cells (B16-F10). To further show the usefulness of multiple cellular targeting in cancer therapies, we have developed a remote loading method to encapsulate DOX inside DMVs. We found that we can load 12% (w/w) DOX inside DMVs, the drug loading efficiency that is comparable to other formulations (such as polymer-based or liposome-based carriers) (Figure 6). In addition, therapeutic studies (Figure 7) show that RGD-DMVs loaded with DOX completely inhibit the tumor growth. Our studies reveal that DMVs may have a great impact on developing bacterium-based cancer therapies for translation.

The major concern is the potential pathogenicity of bacteria in translation. In this study, we used non-pathogenic *Escherichia coli* strain BL21 (*E. coli*-BL21) 59. Most importantly, we exploited nitrogen cavitation approach to eliminate intracellular components to form DMVs 35, thus possibly avoiding the toxicity. Indeed, *in vitro* (Figure S8) and *in vivo* (Figure S10) studies show that DMVs did not appear toxicity to major organs. Although we observed the inflammation response (Figure S9), this mild response is needed to mediate the transport of DMVs across the blood vessel barrier.

In this paper, we have developed the nitrogen cavitation approach to quickly generate bacterial membrane vesicles from *E. coli* BL21, and the vesicles possess intact membrane structures with the size of 200-240 nm in diameter (as shown in Figure 2C-E). Our previous studies show that our nitrogen cavitation can apply to a wide range of cells and may be ready to scale up the production for translation 27, 35. Compared to current cell-based therapeutics, our methods to genetically engineer non-pathogenetic bacteria and to efficiently generate bacterial membrane nanovesicles may offer the potential translation of bacterium-based therapeutics to treat cancer and infectious diseases 37.

## Conclusion

In summary, the studies demonstrate that bacterium-based therapeutics made of DMVs can overcome several physiological barriers existing in TMEs. We have established a pH-gradient method to remotely load DOX inside DMVs by addition of cholesterol to membrane of DMVs. RGD-expression on DMVs demonstrates the enhanced cancer therapy because RGD increases the tumor deposition of DMVs via targeting of multiple cells in tumor tissues. Our data may have a great impact on developments of bacterium-based therapeutics to treat a wide range of cancers. Most importantly, DMVs may be a novel platform to develop therapeutics to treat cancer and infectious diseases because their production and genetical editing are feasible in translation.

## Supplementary Material

Supplementary figures.
