# Supplementary material for: RGD-expressed bacterial membrane-derived nanovesicles enhance cancer therapy via multiple tumorous targeting
Source: Theranostics. 2021 Jan 15;11(7):3301–16. doi: 10.7150/thno.51988 (PMC7847689; doi:10.7150/thno.51988)
Supplement: Supplementary file 1 — Supplementary figures. [file thnov11p3301s1.pdf]

Supplementary Information

RGD-expressed bacterial membrane-derived nanovesicles loaded with doxorubicin enhance cancer therapy via multiple tumor targeting

Jin Gao†, Sihan Wang†, Xinyue Dong, Zhenjia Wang*

Department of Pharmaceutical Sciences, College of Pharmaceutical Sciences and Pharmacy, Washington State University, Spokane, WA 99202, USA

*Correspondence should be addressed to Zhenjia Wang: zhenjia.wang@wsu.edu

† represents the equal contribution to this work.

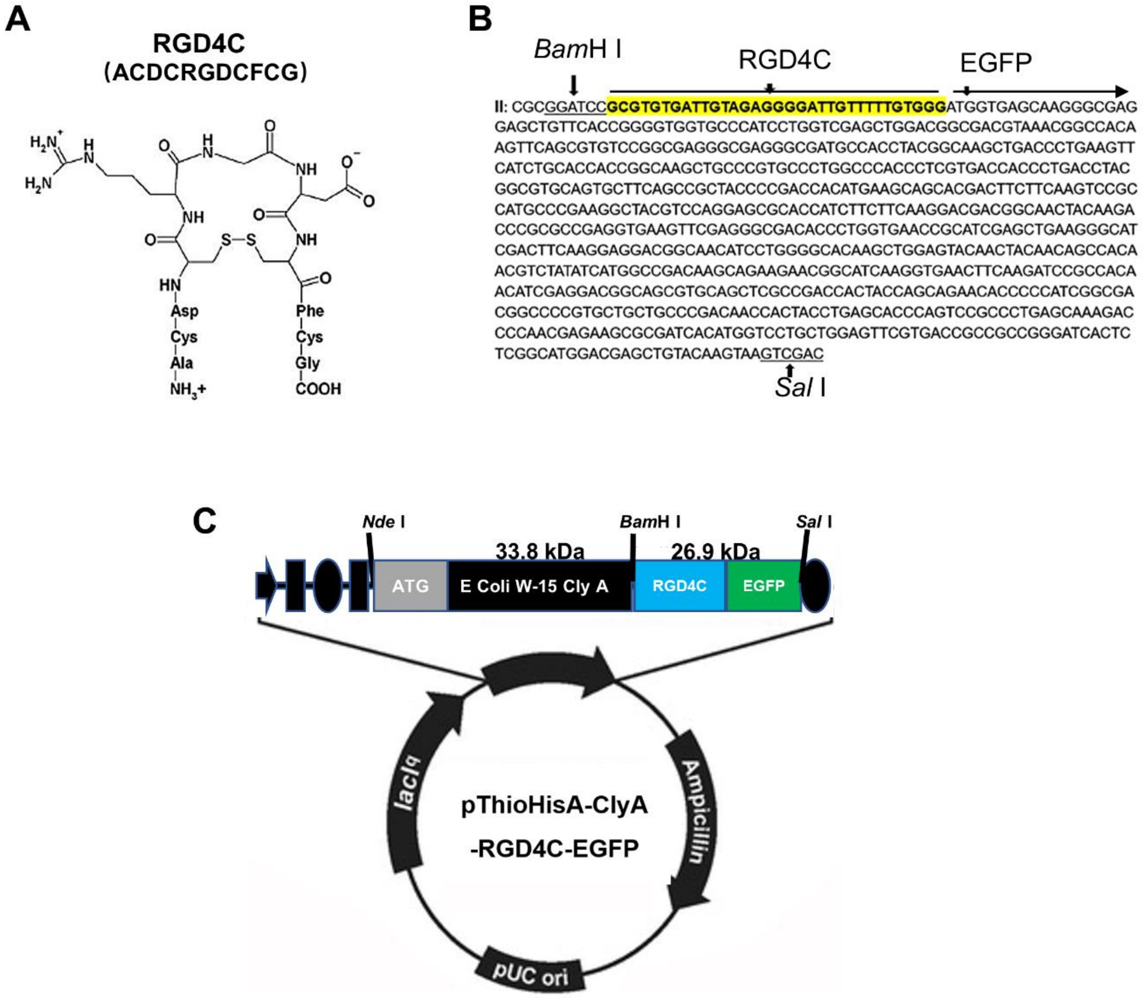

Figure S1. Genetical engineering of a ClyA-RGD4C-EGFP fusion protein and nucleotide sequences of ClyA-RGD4C-EGFP. (A) Chemical structure of RGD peptide. **(B)** Nucleotide sequence of RGD4C-EGFPC. **(C)** Diagram of the recombinant plasmid expressing the fusion protein ClyA-RGD4C-EGFP with the thioredoxin sequence totally replaced by ClyA-RGD-EGFP’s.

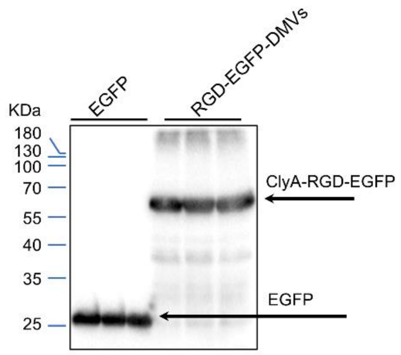

Figure S2. Western blots (WB) of Cly A-RGD-EGFP-DMVs and EGFP. In the experiment, RGD-EGFP-DMVs (20 μg) and the standard EGFP (0.1 μg) were loaded for WB, and 3 times of experiments were shown. Based on the EGFP intensity, we estimated the numner of RGD-EGFP per DMVs.

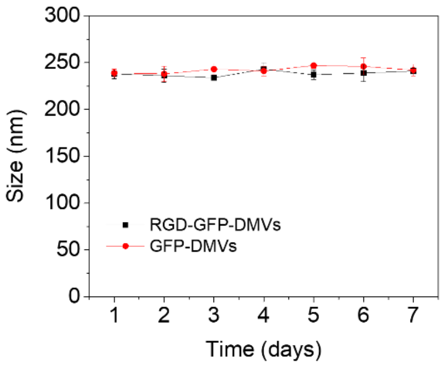

Figure S3. Stability study result of EGFP-DMVs and RGD-EGFP-DMVs. Sizes of EGFP-DMVs and RGD-EGFP-DMVs in serum (10%) at 4ºC were measured by DLS each day for 7 days. Data were expressed as mean±SD, n = 6.

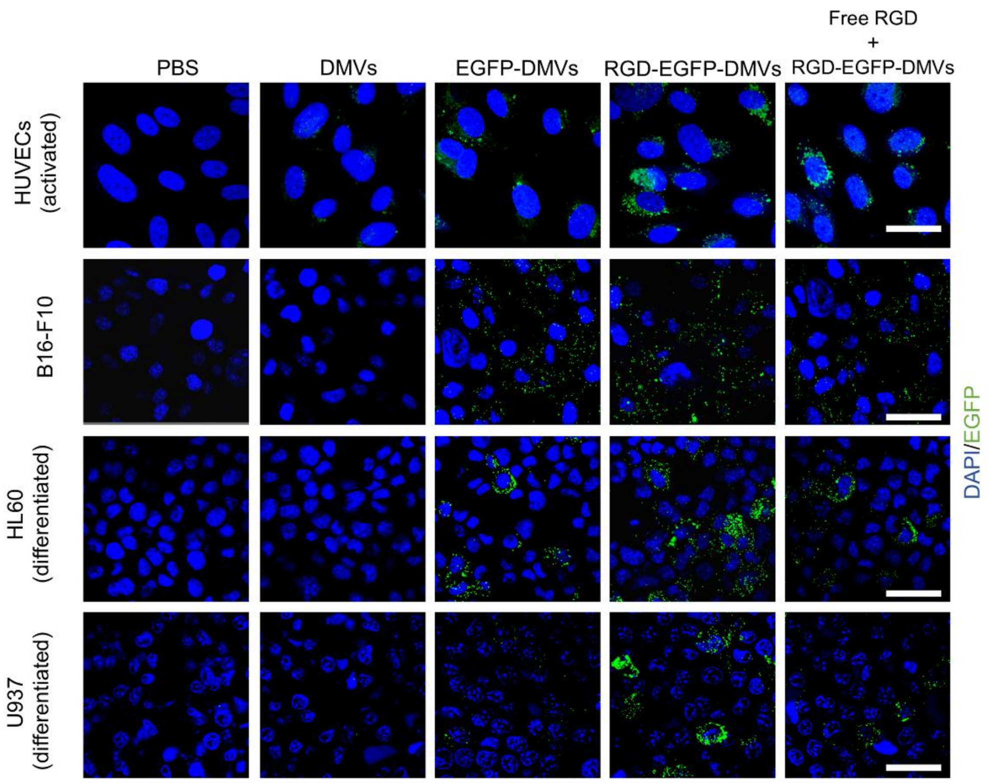

Figure S4. Confocal images of the cellular uptake of DMVs, EGFP-DMVs or RGD-EGFP-DMVs by activated HUVECs, B16-F10 melanoma cells, differentiated HL60 cells (neutrophils like) and differentiated U937 (monocytes like). The cells were incubated with 12 μg DMVs with or without the blocking of free RGD peptides (10 μM) for 60 min at 37°C. Scale bar = 25 μm.

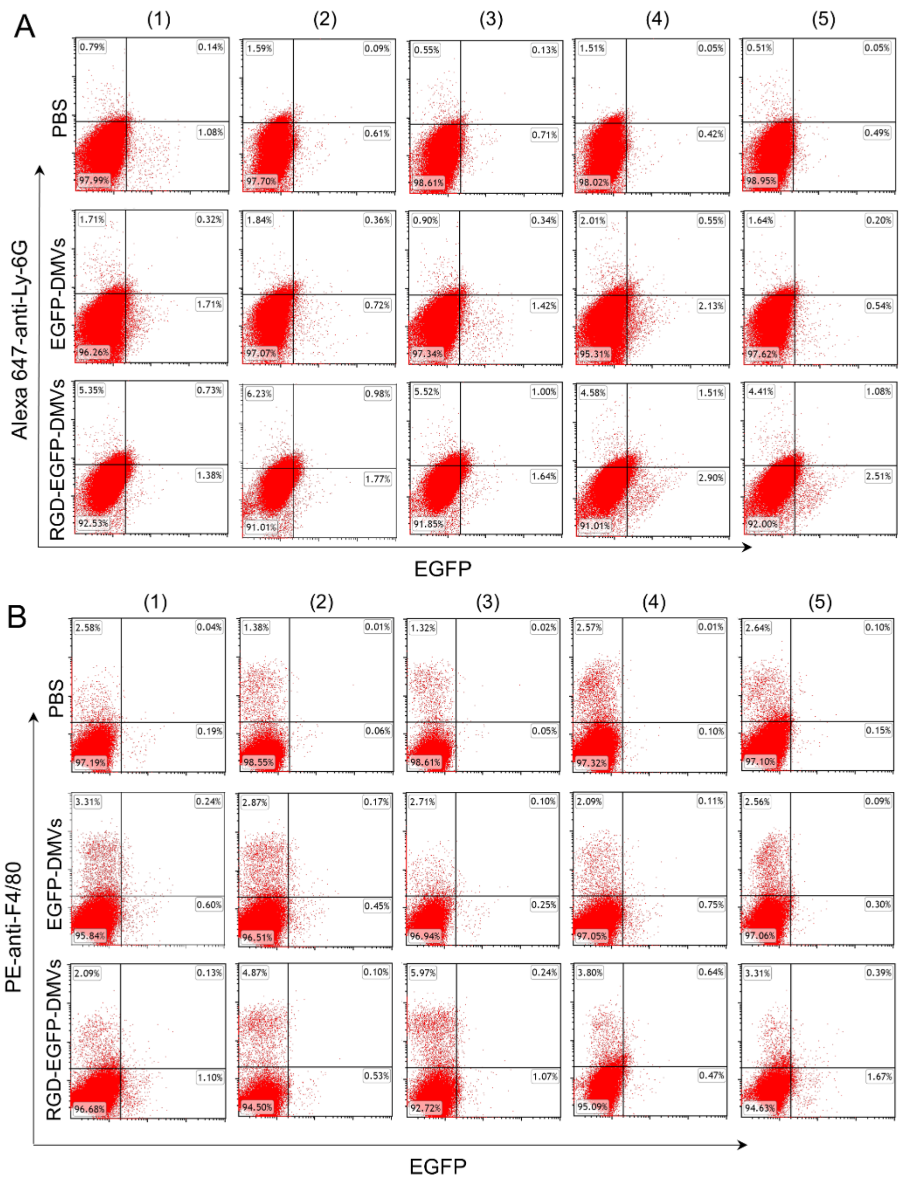

Figure S5. Flow cytometry analysis of neutrophils, monocytes and the total cells containing DMVs in tumor tissues. PBS, EGFP-DMVs and RGD-EGFP-DMVs were given to the melanoma-bearing mice respectively and tumor tissues were collected to get single cell suspensions for flow cytometry. The samples were stained with Alexa 647-anti-LY-6G for neutrophils (A) and PE-anti-F4/80 for monocytes (B), respectively. The number 1-5 represents 5 mice used in the experiments. The gating strategy was established based on the flow cytometry analysis of the single cells isolated from xenograft without DMV injection and cell marker staining.

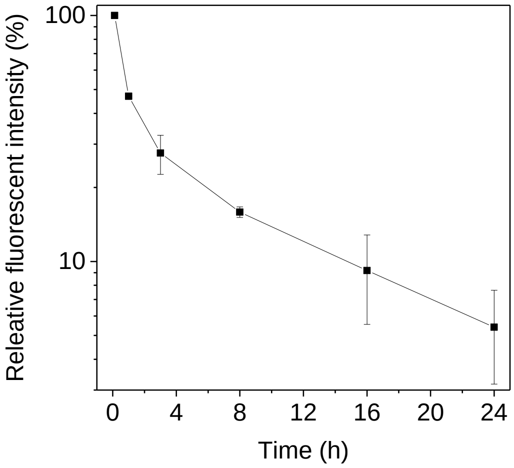

Figure S6. Pharmacokinetics of RGD-EGFP-DMVs in the melanoma mouse model. The plasma fluorescence intensity was measured when the tumor volume was around 50-100 mm^3. The plasma was collected after the animals were iv injected 600 μg of DiR-DMVs and the fluorescence intensity was measured on a plate reader (n = 3, mean ± SD).

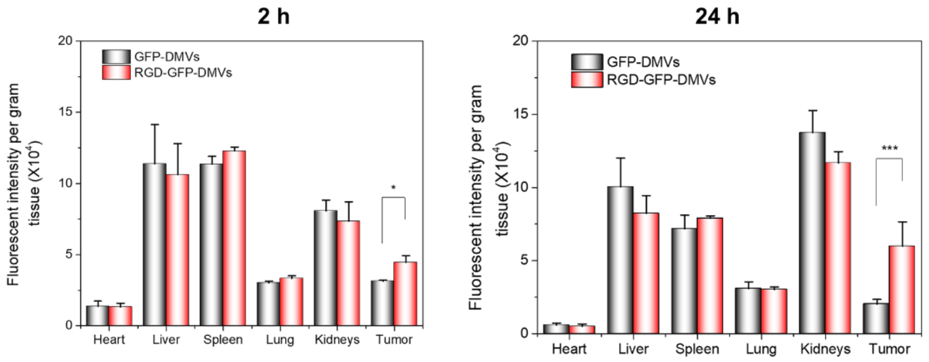

Figure S7. Biodistribution of RGD-EGFP-DMVs. 2 h and 24 h after DiR-RGD-EGFP-DMVs and DiR-EGFP-DMVs (equal fluorescent intensity, both at around 10 mg/kg in proteins) were *i.v.* administered to tumor bearing mice, the fluorescent signals were measured after tissues were homogenized (n = 3, mean ± SD).

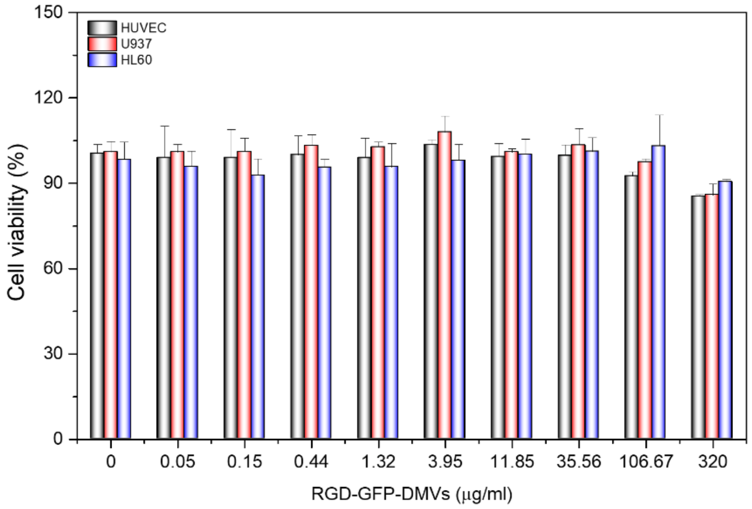

Figure S8. Toxicity of RGD-EGFP-DMVs on HUVECs, U937 and HL60 cells. 10 000 cells were incubated with RGD-EGFP-DMVs for 24 hours and 10 μl MTS stock solution was added into each well. 4 hours later, the absorbance at 490 nm of the wells was measured on a plate reader (n = 4, mean ± SD).

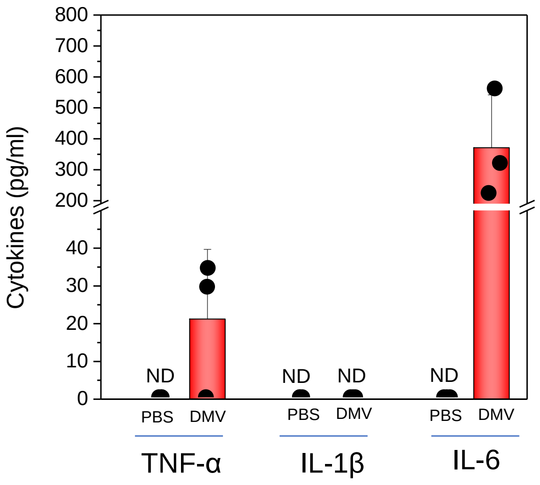

Figure S9. Systemic immune responses of RD-EGFP-DMVs in the tumor mouse model. Cytokines including TNF-α, IL-1β and IL-6 were measured by ELISA 24 h after administration of DMVs (n = 3, mean ± SD) at 600 μg of DiR-DMVs. The tumor volume was around 50-100 mm^3.

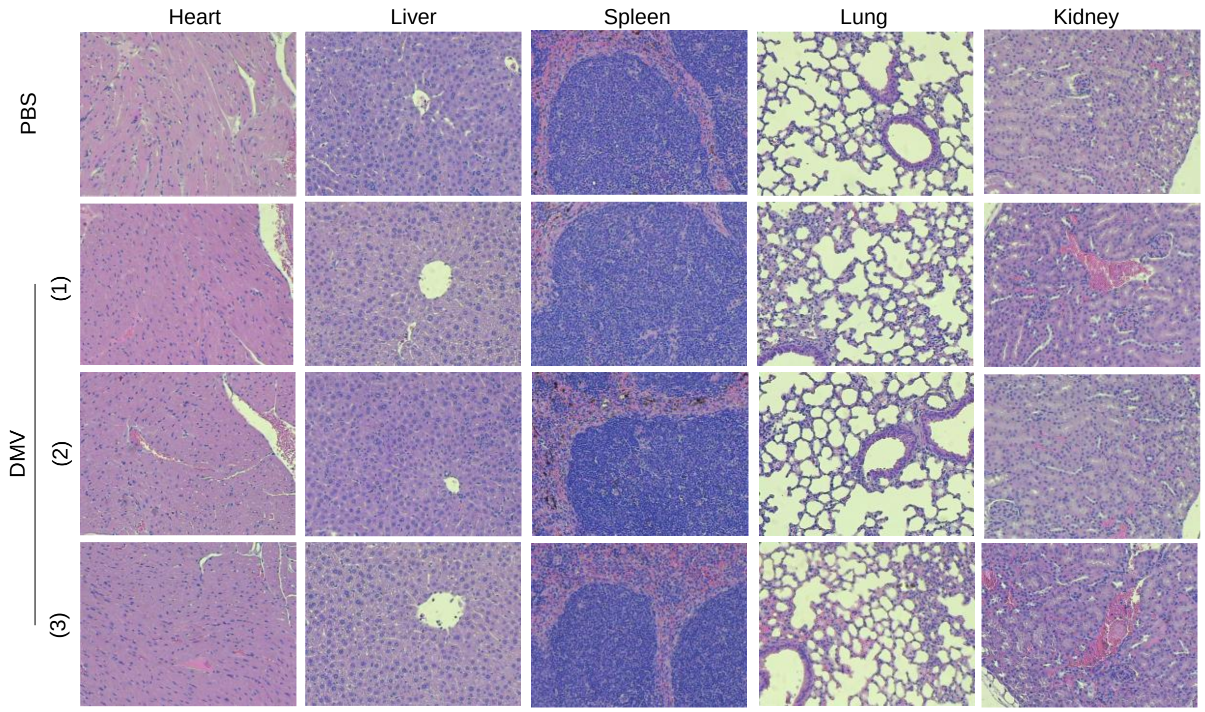

Fig. S10 Histology of heart, liver, spleen, lung and kidney in the B16-F10 mouse after the treatments by PBS or 600 μg of DMVs. The sections were stained by H&E and the images were taken using a Leica DM2500 LED microscope (Original magnification, 10 × 20; n = 3). No visible histological difference between the PBS group and the group of DMVs. There were triplet experiments for the group of DMVs.
